# Distribution of honey bee mitochondrial DNA haplotypes in an Italian region where a legislative act is protecting the *Apis mellifera ligustica* subspecies

**DOI:** 10.1038/s41598-024-71233-5

**Published:** 2024-09-04

**Authors:** Valeria Taurisano, Anisa Ribani, Dalal Sami, Kate Elise Nelson Johnson, Giuseppina Schiavo, Valerio Joe Utzeri, Samuele Bovo, Luca Fontanesi

**Affiliations:** Animal and Food Genomics Group, Division of Animal Sciences, Department of Agricultural and Food Sciences, University of Bologna, Viale Giuseppe Fanin 46, 40127 Bologna, Italy

**Keywords:** Apiculture, Beekeeping, Conservation genetics, Genetic integrity, Mitotype, Ecology, Genetics

## Abstract

The conservation of the genetic integrity of *Apis mellifera* subspecies has emerged as an important objective. In 2019, the Emilia-Romagna region became the first Italian regional authority to issue a law specifically addressing the protection of the native *Apis mellifera ligustica* subspecies. In this study we analysed a highly informative portion of the mitochondrial DNA (mtDNA), widely used for assessing genetic diversity of honey bee populations. By analysing 1143 honey bees sampled after the introduction of this law, we provided a snapshot of the distribution of mtDNA haplotypes in this region. The two most frequent mtDNA haplotypes were C1 (characteristic of *A. m. ligustica*) and C2 (characteristic of *A. m. carnica*), reported in 86.5% and 11.0% of the analysed bees, respectively. About 1.3% and 1.1% of the analysed bees carried mtDNA haplotypes of the A and M lineages (haplotypes A1a, A1e, A4, A26, A65 and two novel ones, A2w and A6a; M3, M3a, M4 and M79). Continued genetic monitoring will be important to assess the impact of this regional law over the coming years. Based on the obtained results, we recommend a more stringent policy to prevent the erosion of the genetic integrity of the native subspecies *A. m. ligustica*.

## Introduction

*Apis mellifera* Linnaeus, 1758, commonly known as the western honey bee or simply honey bee, is a highly polytypic managed pollinator species. About 30 honey bee subspecies have been described thus far, originally spread throughout its native regions, which include Europe, Africa and Western Asia^1–4^. Local honey bee populations and subspecies are considered well adapted to their native environments, and conserving their genetic integrity is important for the long-term sustainability of beekeeping activities and related ecosystem services, including pollination in agricultural production and agroecological systems^5–7^.

The description and, in turn, the classification of the different *A. mellifera* subspecies have utilised various types of information, including the native geographic distribution, behavioural and morphometric features, allozyme polymorphisms, and, more recently, polymorphisms at the mitochondrial DNA (mtDNA) and nuclear genome levels, probed using a variety of approaches^1–4,8,9^. Despite several inconsistencies in this classification across types of information, methods and authors, the described subspecies have been grouped into four or five evolutionary lineages, reflecting, in some cases, the problems mentioned for the subspecies assignment: lineage A, the African lineage (mainly spread in African regions and in South Europe, including the Iberian Peninsula and several Mediterranean islands, such as Sicily and Malta); lineage C (mainly spread in South-Eastern European regions, including the Italian Peninsula); lineage M (mainly spread in West and North Eurasia, from the British Isles through most of continental Europe, to the Ural and some areas in Central Asia, but also including the Iberian Peninsula); lineage O (mainly distributed in the North of the Middle-East); and the recently proposed lineage Y, grouping North-Eastern African populations^2,3,10–15^. The geographical distribution of these lineages includes many contact regions where admixed honey bee populations/subspecies have been established and that, in some cases are challenging to distinguish between them using one or another type of information^16^.

Mitochondrial DNA (mtDNA) has been widely used to evaluate the phylogenetic relationships within the *A. mellifera* species. The most frequently targeted mtDNA region for these studies is the highly polymorphic region spanning the tRNAleu and COII genes (originally named COI-COII intergenic region), traditionally investigated using the PCR–RFLP assay with the *Dra*I restriction enzyme (*Dra*I test)^3,17,18^. The polymorphisms in this region stem from the combination of a short nucleotide unit of about 50–70 bp (referred to as the P unit, which can take four major forms: P0, P, P1 and P2, which differ from each other due to insertions and deletions), and a long sequence element of about 190–200 bp (referred to as the Q unit, which can exist in one to five tandem repeated copies)^3,17,18^. Honey bee mtDNA evolutionary lineages can be distinguished by the absence of the P unit, which is a characteristic of the C lineage (carrying only one Q unit), and by the presence of the P sequence in all other lineages. These lineages are further distinguished by various P variants and variability in the number and sequence of the Q unit^17^. Sequencing the amplified DNA fragments can overcome some limitations of the *Dra*I test, which cannot capture all variability in the tRNAleu-COII region^19,20^. The sequence information obtained can then be assigned to an mtDNA haplotype (or haplogroup or mitotype) by comparing it to corresponding sequences from previous studies [e.g.^15,20–23^]. A few nomenclature systems were proposed and refined during the last decade aimed to distinguish haplotype variants based on the different *Dra*I band patterns and sequence data^19,20^.

Numerous *A. mellifera* populations in their native regions have been genetically characterized using mtDNA analyses to evaluate the genetic integrity of local honey bee genetic resources, including subspecies and ecotypes (e.g.^15,19,20,23–25^). Most of these studies, primarily focused on European regions, have shown varying degrees of introgression and admixture between different *A. mellifera* lineages, subspecies and populations^5,15,23,26–35^. This is mainly caused by extensive trading of queen bees and the transhumance of colonies, which, in several cases, are practices necessary to replace lost colonies or counterbalance the effects of climate change on nectar availability^36–43^. The non-native genetic pools usually derive from *A. m. ligustica*, *A. m. carnica* and hybrid Buckfast lines for which well-established breeding programmes can provide queen bees that are commonly distributed across the world. In particular, the widespread use and commercial distribution of *A. m. ligustica* are attributed to its high productivity, adaptability to a wide range of environments, docility, and prolificity^1^.

Italy, including its Peninsula and the main islands of Sardinia and Sicily, is geographically positioned at the crossroads of the natural distribution of four *A. mellifera* subspecies from different lineages^1,24,25,44^: *A. m. ligustica*, belonging to the C lineage and originating from the Italian Peninsula, mainly carrying the C1 mitotype but also reported to carry M7 mitotypes, although the nomenclature of this haplotype needs some refinements^45^; *A. m. siciliana*, native to Sicily and carrying mitotypes of the A lineage; *A. m. mellifera*, belonging to the M lineage and therefore carrying M mitotypes, originally distributed in small areas of the Western Alpine arch; and *A. m. carnica*, belonging to the C lineage, with natural populations in the North-East part of Italy, near the border with Austria and Slovenia, and mainly carrying the C2 mitotype^25,47–50^. We recently reported an updated distribution map of the main *A. mellifera* mtDNA lineages in Italy based on the analysis of honey bee DNA found in the honey^48,50^.

**Fig. 1:**
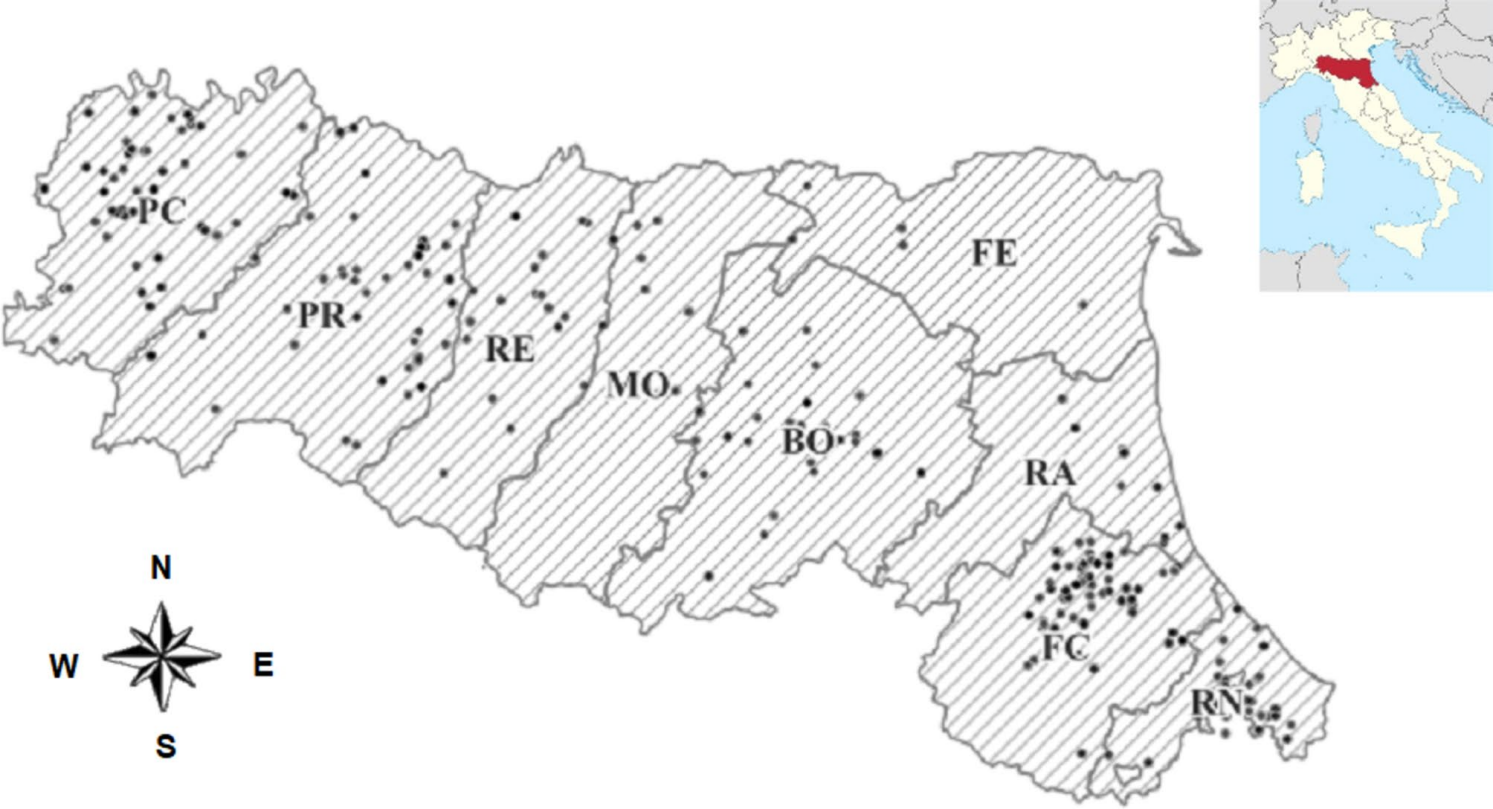
Geographical distribution of the apiaries of the Emilia-Romagna region (22,510 km^2^) from which honey bees were sampled. Province abbreviations and administrative borders are highlighted. The correspondence between province name and their abbreviation is reported in Table 1. The subset indicates the position of region in the Italian map.

A few national or regional legislative acts or initiatives have been introduced in Europe, including Italy, to preserve the genetic integrity of native honey bee subspecies^6,24,51,52^. In Italy, for example, the Emilia-Romagna region (located in the north of Italy, just south of the Po River; Fig. 1) has been the first regional authority to issue a regional law, specifically addressing this question: regional law n. 2 of the 4th of March 2019 (Regione Emilia Romagna, 2019). Article 7 of this regional law, entitled “Tutela dell’*Apis mellifera ligustica*”, focuses on the protection of this subspecies, prohibiting the breeding and introduction of subspecies different from *ligustica* in the region, and providing the opportunity to designate reproduction areas^51^. The significance of this regional law stems from the fact that this region is home to one of the largest concentrations of queen breeding activities for the native subspecies *A. m. ligustica* in Italy, and possibly in the world.

In this study, we investigated the diffusion and distribution of mtDNA haplotypes in the honey bee population of the Emilia-Romagna region, by analysing honey bees sampled in the years 2020, 2021 and 2022, just after the introduction of the regional law that protects *A. m. ligustica*. We hypothesised that the effects of the law may not yet be evident, as only one or a few years passed since its publication. Therefore, the observed frequency of mtDNA haplotypes in the honey bee population of the Emilia-Romagna region could serve as a baseline for monitoring the trend in the frequency of *A. m. ligustica* specific mtDNA haplotypes over the next few years, reflecting the effectiveness of the law’s enforcement.

## Methods

### Honey bee samples

This study was conducted using 1143 honey bees (workers, pupae, or larvae) sampled in 2020 (n. 94), 2021 (n. 588), and 2022 (n. 461). Each bee was collected from a different colony of apiaries located in the Emilia Romagna region (Northern Italy), owned by a total of 32, 114 and 60 beekeepers over three years, respectively. Colonies were sampled from either the same or different apiaries, with an average of approximately four colonies per apiary and beekeeper. The geographical location of these apiaries is shown in Fig. 1. Table 1 summarizes the sampled honey bees divided by administrative province of the Emilia-Romagna region and by year. Bees were placed in absolute ethanol and stored at + 4 °C until DNA extraction.

**Table 1:** Number of analysed honey bees by Administrative Province of the Emilia-Romagna region and by year of sampling.

Administrative province	Years of sampling			Total number
	2020	2021	2022	
Piacenza (PC)	13	48	115	176
Parma (PR)	19	107	87	213
Reggio Emilia (RE)	5	36	25	66
Modena (MO)	3	30	0	33
Bologna (BO)	29	65	56	150
Ferrara (FE)	11	0	5	16
Ravenna (RA)	4	67	6	77
Forlì – Cesena (FC)	3	166	145	314
Rimini (RN)	7	69	22	98
Total number	94	588	461	1143

### DNA extraction, PCR and sequencing

DNA was extracted from a portion of the head of each honey bee. The extraction was carried out using the Wizard Genomic DNA Purification Kit (Promega, Promega Corporation, Madison, WI, USA) following the manufacturer’s protocol for animal tissues. DNA integrity was evaluated by electrophoresis on TBE1X 1% agarose gels stained with 1XGelRed Nucleic Acid Gel Stain (Biotium Inc., Haward, CA, USA) and was quantified using a Nanophotometer P-330 instrument (Implen GmbH, München, Germany). Honey bee DNA was amplified using the primer pair designed by Garnery et al*.*^8^ (E2, forward: 5’-GGCAGAATAAGTGCATTG-3’; H2, reverse: 5'-CAATATCATTGATGACC-3’). These primers amplify a portion of the intergenic non-coding region of the mtDNA located between COI and COII that contains the tRNA-Leu sequence (tRNALeu-COII) and allows the discrimination between different *A. mellifera* mtDNA haplotypes. PCR was performed using an Applied Biosystem SimpliAmp Thermal Cycler (Thermo Fisher Scientific Inc., Waltham, MA, USA) in a total reaction volume of 14 µL. The reaction mix contained: 2X Kapa Hifi HotStart ReadyMix PCR kit (Kapa Biosystems, Boston, MA, USA); 20–50 ng of template DNA; 10 pmol of each primer. The PCR amplification profile included an initial denaturation step at 95 °C for 5 min, followed by 35 cycles of alternate temperatures (30 s at 95 °C, 30 s at 54 °C; 30 s at 72 °C) and by a final extension step at 72 °C for 5 min. The obtained amplicons were electrophoresed on a 2.5% agarose gels in TBE1X buffer, stained with 1X GelRed Nucleic Acid Gel Station (Biotium Inc.).

PCR products were purified with a standard isopropanol precipitation-purification protocol before Sanger sequencing. Sequencing reactions obtained using both primers were loaded on an ABI3100 Avant Genetic Analyzer Sequencer (Applied Biosystems, Foster City, CA, USA) following the chain termination protocol of the BrightDye terminator cycle sequencing kit (NIMAGEN, Nijmegen, the Netherlands). Novel mtDNA sequence haplotypes were confirmed with a second DNA extraction, PCR, and subsequent Sanger sequencing, carried out as described above.

### Sequence data analyses and geographical distribution of mtDNA haplotypes

Electropherograms were visually inspected using MEGA XI software^53^. MEGA XI was also used to build a multiple alignment using reference sequences of *A. mellifera* mtDNA retrieved from the GenBank database (http://www.ncbi.nlm.nih.gov/nucleotide/, accessed on the 20th of January 2024). BLASTN tool (http://www.ncbi.nlm.nih.gov/BLAST/, accessed on the 20th of January 2024) was used to compare the obtained sequences and validate their assignment to different *A. mellifera* mtDNA haplotypes. Geneious Prime 2022.0.1 software (https://www.geneious.com) was used to obtain the in silico* Dra*I profile according to the guidelines of the previously established *Dra*I mtDNA test^8^.

Phylogenetic analyses, including one sequence for each mtDNA haplotype identified in this study and corresponding representative sequences retrieved from GenBank/ENA databases and derived from previous studies (Supplementary Table S1), were run using MEGA XI software^53^ and were based on a Maximum Likelihood (ML) model and on a Neighbour-Joining computed using the Maximum Composite Likelihood method (NJ-MCL), with trees obtained with 1000 bootstrap replicates. The ML tree was obtained using the General Time Reversible base substitution model considering Gamma and Invariable sites (GTR + G + I). In this analysis, initial trees for the heuristic search were obtained automatically by applying Neighbour-Joining and BioNJ algorithms to a matrix of pairwise distances estimated using the MCL approach and subsequently by selecting the topology with superior log likelihood value. A discrete Gamma distribution was used to model evolutionary rate differences among sites (+ G, parameter = 0.5666) while the rate variation model allowed for some sites to be evolutionarily invariable ([+ I], 26.06% sites). Specifically, the GTR nucleotide substitution model was selected as it encompasses all transition and transversion substitutions and allows for unequal base frequencies. Additionally, this model is considered the best choice for Maximum Likelihood phylogenetic reconstruction. For the NJ-MCL tree, the evolutionary distances were computed using the Maximum Composite Likelihood method and the rate variation among sites was modelled with a Gamma distribution (shape parameter = 1). To simplify the phylogenetic representation derived from both trees, branches distinguished by less than 20% of the bootstrap replicates were collapsed together and all ambiguous positions were removed for each sequence pair using the pairwise deletion option.

Haplotype diversity (*H*), considered as the probability that two randomly sampled alleles are different, was calculated according to the following formula:

$H=\frac{N}{N-1} \left(1- {\sum }_{i}{x}_{i}^{2}\right),$ where N is the sample size of the population and *x*_*i*_ is the relative haplotype frequency of each haplotype within the analysed population^54^. Fisher’s exact test was used to assess whether there was any population differentiation in terms of mtDNA haplotypes across time.

Software QGIS 3.30 (http://www.qgis.org, accessed on the 20th January 2024) was used to produce a geographical representation and density map of the distribution of the mtDNA haplotypes over the whole Emilia-Romagna region. The density map was obtained using the *heatmap* plugin of this software. The density was calculated based on the number of points in a location, with larger numbers of clustered points resulting in larger values. This approach, heatmaps allow the identification of “hotspots” and clustering of points. Logistic regression was used to test relationships between the distribution of a specific mtDNA lineage or haplotype (coded as binary variable: presence/absence of a mitotype) across the longitudinal geographic coordinates of the Emilia-Romagna region.

## Results

### Honey bee mtDNA haplotypes

Amplified fragments and sequences were obtained from all 1143 sampled honey bees. Amplicon size ranged from about 550 to 900 bp, as determined from agarose gel electrophoresis, and then confirmed from the analysis of the obtained sequencing electropherograms.

After sequencing, based on various polymorphisms of the tRNALeu-COII region, we identified a total of 14 different mtDNA haplotypes, whose structure and *Dra*I restriction sites were determined in silico and are shown in Supplementary Figure S1. These haplotypes were grouped into three main mtDNA lineages: A, C, and M. Twelve of these mtDNA haplotypes have already been reported by previous studies; two are novel haplotypes, that, as far as we know, are described in this study for the first time.

By far, the two most frequent mtDNA haplotypes identified were C1 and C2, reported respectively in 86.5% and 11.0% of the analysed honey bees. A total of 989 out of 1143 honey bee mtDNA sequences showed 100% identity with haplotype C1 of *A. m. ligustica* (accession number NC_001566). Additional 124 mtDNA sequences had 100% identity with haplotype C2 of *A. m. carnica* (accession number NC_061380.1) and two were identical to the C2c haplotype (MN_250878.1), originally assigned to *A. m. carnica* (accession number MN_250878.1). This sequence has a deletion of one cytosine at position g.3431 and shared two nucleotides typical of *A. m ligustica* at position g.3632 (T) and g.3767 (C), based on the reference sequence NC_001566 of *A. m. ligustica* mtDNA. The C1 and C2 haplotypes are considered specific haplotypes of the *A. m. ligustica* and *A. m. carnica* subspecies, respectively^25,33,48,49,55,56^.

About 1.3% (15 sequences) and 1.1% (13 sequences) of the total analysed sequences showed identity with different mtDNA reference sequences belonging to the A and M lineages, respectively. Among the mtDNA sequences assigned to the A lineage, 12 had 100% identity with haplotypes already described in *A. m. intermissa* and *A. m. iberiensis* subspecies, namely haplotypes A1a [accession number KX463739^20^; for five sequences], haplotype A1e [MW677198^21^; for three sequences], haplotype A4 (EF033650^57^; for two sequences), haplotype A26 [EF033651^57^; for one sequence] and haplotype A65 [accession number MW677213^21^; for one sequence]. Three sequences belonging to the A lineage were from two novel mtDNA haplotypes (named as A2w and A6a, according to the accepted nomenclature, including *Dra*I restriction sites). Detailed descriptions of these two mitotypes are reported below. The 13 mtDNA sequences of the M lineage had 100% identity with five haplotypes already described in *A. m. mellifera* and *A. m. iberiensis* subspecies: haplotypes M3 [FJ743636.1^58^; for one sequence], haplotype M3a [KX463884^20^; for eight sequences], M4 [EF033656^57^; for three sequences] and haplotype M79 [KX463882^20^; for one sequence]. A summary of the identified mtDNA haplotypes, including matched sequences already reported by others, is provide in Supplementary Table S1.

**Fig. 2:**
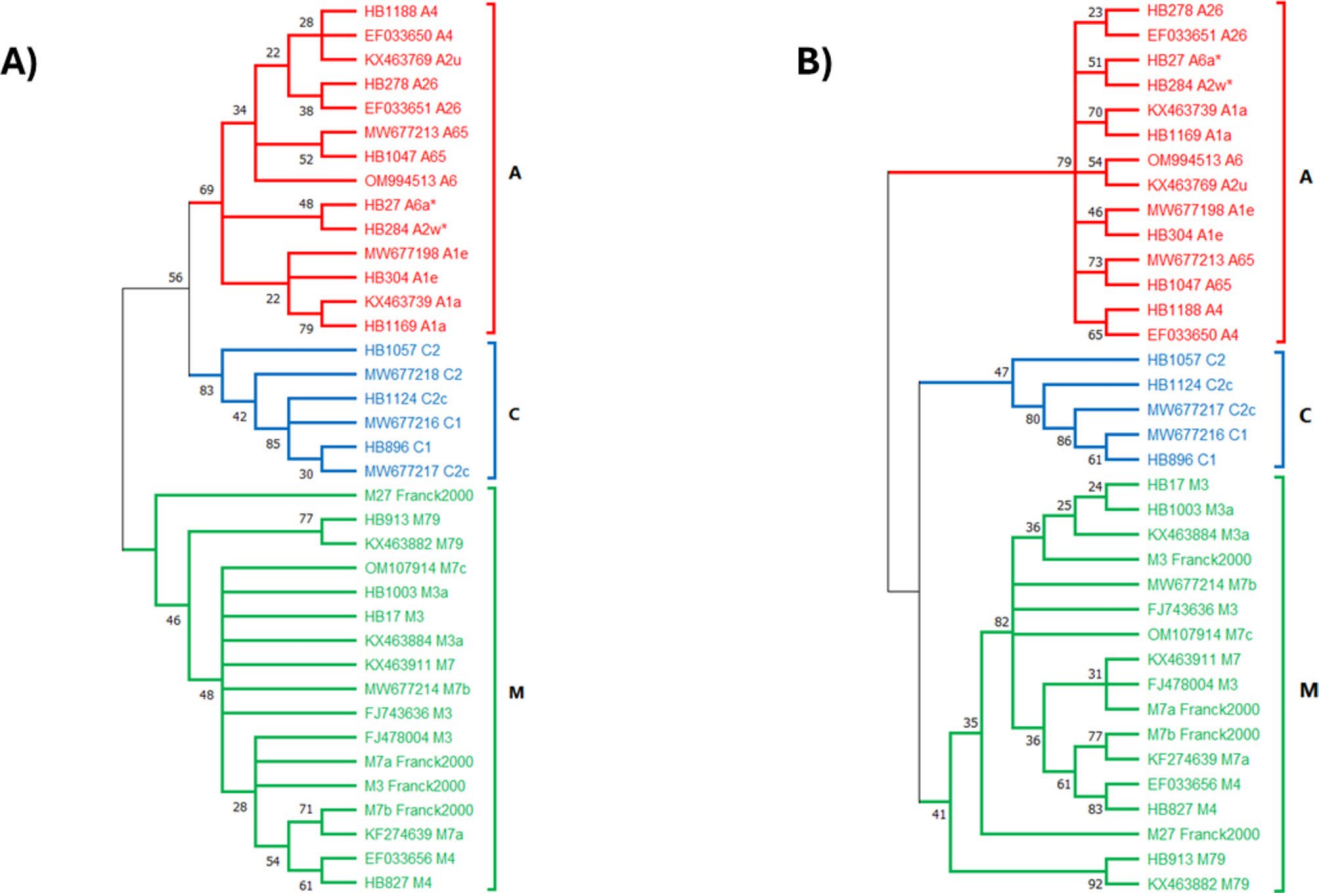
(**A**) Maximum Likelihood (ML) and (**B**) Neighbor-Joining Maximum Composite Likelihood (NJ-MCL) phylogenetic trees obtained from the tRNALeu-COII mtDNA region with 1000 bootstrap replicates. The percentage of replicate trees in which the associated taxa clustered together in the bootstrap test are shown next to the branches. Nodes with percentage of replicates < 20% were collapsed (for example, HB1057 C2 and MW677218 C2 that are separated in the ML tree have been collapsed in the NJ-MCL tree). Sequences retrieved from GenBank are reported with the respective accession number, followed by the haplotype name; sequences obtained from samples analysed in this study are reported with abbreviation HB followed by the assigned laboratory number and the name of the assigned haplotype. Haplotypes derived from Franck et al*.*^25^ are indicated with the haplotype name, as reported in the figures of the original article, followed by the acronym “Franck2000”. The two novel haplotypes are marked with an asterisk.

Figure 2 reports the phylogenetic relationships of the 14 mtDNA haplotypes in an unrooted Maximum Likelihood (ML) tree and in a Neighbour-Joining Maximum Composite Likelihood (NJ-MCL) tree, including additional mtDNA sequences representing examples of mtDNA retrieved from GenBank/ENA databases reported by other studies (Supplementary Table S1).

Phylogenetic analyses showed the expected tree topology, with sequences from the same evolutionary lineage clustering together with statistically supported nodes. The M lineage formed a monophyletic clade, while the A and C lineages formed two sister groups with strongly supported nodes. In the phylogenetic tree, the C lineage appeared as a robust clade, despite the clustering of the C1 and C2c haplotypes in the ML tree due to the sharing of two nucleotides in the COII gene coding region (g.3632 T and g.3767C). The phylogenetic positions of several M3 and M7 haplotypes within the M lineage remain unresolved. Notably, the M7a and M7b haplotypes, previously indicated to be present in *A. m. ligustica* subspecies^25^, clustered with the M4 haplotype, with strong bootstrap support. Additionally, the A lineage formed a distinct clade with some unresolved subclusters, where the two novel haplotypes (A2w and A6a) identified in this study clustered together with high bootstrap support in both phylogenetic trees (Fig. 2).

### Detailed characterisation of the novel mtDNA haplotypes

The two novel haplotypes have been named A2w and A6a, adopting the criteria proposed by Chávez-Galarza et al*.*^20^. The characteristics of these two novel haplotypes and their variant positions are shown in Supplementary Figure S1 and S2. These haplotypes have one P0 element of 68 bp and two Q elements. Haplotype A2w contains two restriction sites for *Dra*I (TTTAAA). The in silico digestion of this sequence predicts a band pattern of 47/108/674 bp, similar to that of the A2 haplotypes^12,20^. Haplotype A6a contains four restriction sites for *Dra*I and the in silico digestion with this enzyme predicts a profile with fragments of 47/108/63/191/420 bp, similar to that of the A6 haplotypes^20^. The difference in the number of the restriction sites between these two haplotypes is due to two T/A variants at positions 219 of our sequence and 420 of the mtDNA sequence which remove two *Dra*I restriction sites (TTTAAA to TTTTAA). The sequences of these two novel haplotypes have been deposited in ENA (https://www.ebi.ac.uk/ena) within the project number PRJEB67526 and are associated with the accession numbers OY748522 and OY748523. Both novel haplotypes were identified in apiaries located in the province of Piacenza. Haplotype A2w was identified from two different individual bees sampled in 2021 and belonging to two apiaries of the same beekeeper. Haplotype A6a was identified in one honey bee sampled in 2020.

### Haplotype diversity, geographic and across time distribution of the mtDNA haplotypes

**Fig. 3:**
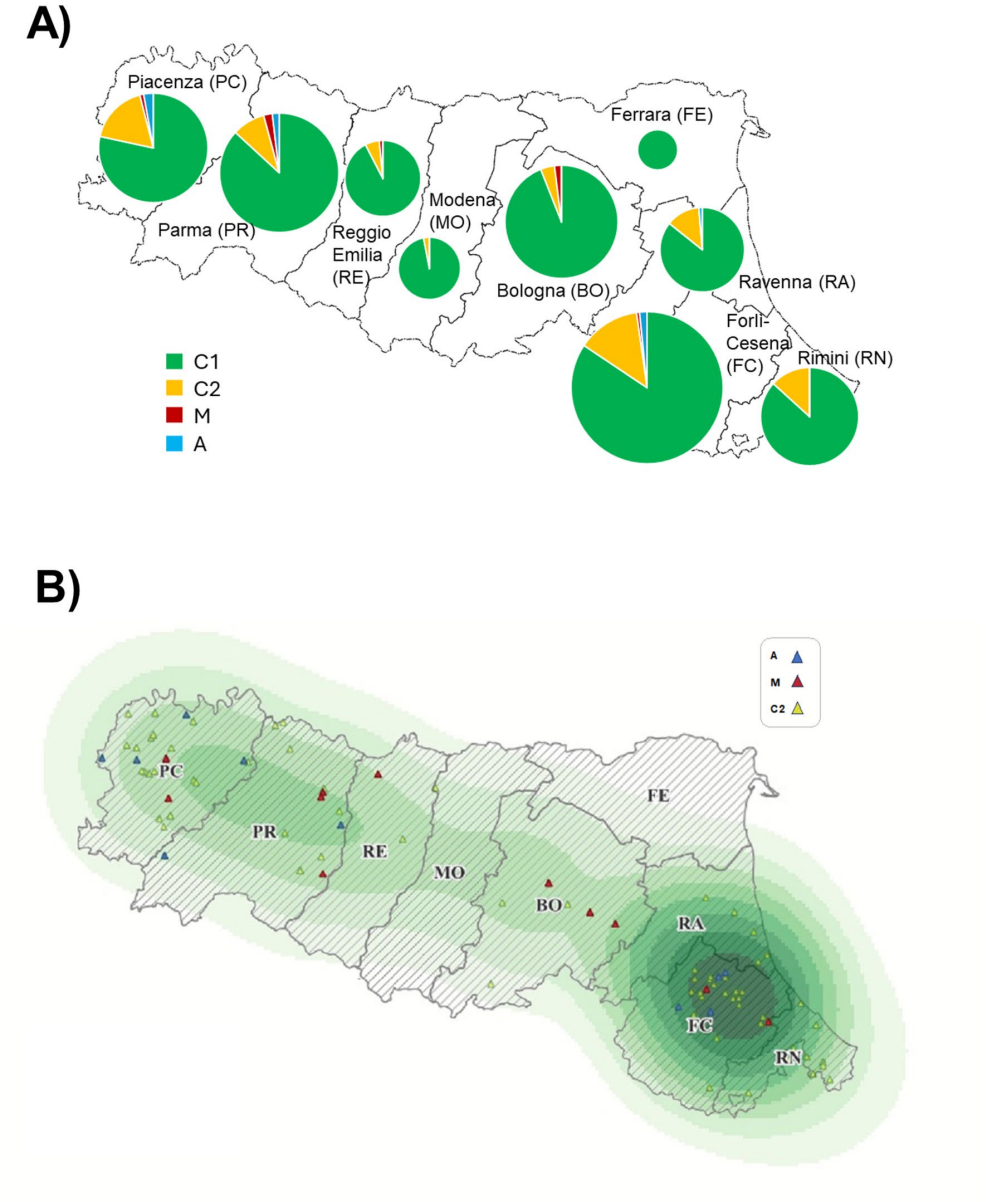
Distribution of the mtDNA haplotypes in the Emilia-Romagna region across the three years of sampling. (**A**) Frequency of the C1, C2, M and A haplotypes in the different administrative provinces. The chart size is proportional to the number of honey bees analysed for each administrative province. (**B**) Density map of the analysed honey bees carrying the C1 mtDNA haplotype. The same map reports information of the geographical position of the apiaries from which some honey bees carried the A (blue triangle), C2 (yellow triangle), or M (red triangle) mtDNA haplotypes.

The frequencies of the mtDNA haplotypes dissected by year of sampling is shown in Table 2. Frequencies did not show any statistically significant differences across the three years of sampling (*P* > 0.20). Figure 3A shows the frequency of the different mitotypes distinguished by administrative provinces of the Emilia-Romagna region, considering all three years of sampling. The number of different haplotypes distinguished by year of sampling and administrative province is reported in Supplementary Table S2. Figure 3B displays the Emilia-Romagna region density map of the distribution of the C1 mtDNA haplotype. The same map includes the geographical position of the apiaries from which some honey bees carrying C2, A or M haplotypes were identified. The A lineage was identified in five administrative provinces (Forlì-Cesena, Piacenza, Parma and Ravenna) and the M lineage was found in five administrative provinces (Bologna, Forlì-Cesena, Piacenza, Parma and Reggio Emilia) across the years of sampling. Logistic regression analysing the longitudinal positions of the apiaries across Emilia-Romagna—a region in northern Italy spanning about half of the country’s longitudinal distance—revealed no significant distribution patterns for the mtDNA haplotypes A, M, and C2 (χ^2^ = 1.724; *P*-value = 0.189), indicating their random distribution throughout the area.

**Table 2:** Number of the analysed honey bee samples per year with the identified mitotype and their frequencies.

		mtDNA haplotypes^1^			
Year	N. of honey bees	C1	C2	M	A
2020	94	80 (0.860)	9 (0.090)	3 (0.040)	2 (0.010)
2021	588	511 (0.869)	65 (0.111)	6 (0.010)	6 (0.010)
2022	461	398 (0.863)	52 (0.113)	4 (0.009)	7 (0.015)
Total	1143	989 (0.866)	126 (0.110)	13 (0.011)	15 (0.013)

^1^Number and, within brackets, frequency of the analysed honey bees carrying different mtDNA haplotypes: the C2 column includes the C2 and C2c haplotypes; the M column includes the M3, M3a, M4, and M79 haplotypes; the A column includes the A1a, A1e, A2w, A4, A6a, A26, and A65. Detailed information is reported in Supplementary Table 2.

The overall Haplotype Diversity (*H*), considering all 1143 analysed honey bee samples, was 0.239, a relatively low level which reflects the high frequency of the C1 haplotype in the region.

## Discussion

Emilia-Romagna is the first Italian regional administrative authority that has recently issued a specific regional law aimed at favouring *A. m. ligustica*, considered the native subspecies^51^. In this study we analysed a large number of honey bee samples to monitor the distribution and diffusion of mtDNA haplotypes of the *A. mellifera* populations managed in this region. The study covers a short time window based on three years of sampling (2020, 2021 and 2022), which may represent in practice a single population genetic snapshot, as no differences between mtDNA haplotype frequencies were evident between the years. This time window may not capture any relevant effects of the regional law as it was issued only in 2019. Therefore the results obtained can be considered a starting point for monitoring its effects. It is also worth mentioning that currently no specific protocols or procedures have been officially established in the region to monitor the application of the law, which may have limited the enforcement of the law in recent times.

Considering the relatively small area from which we collected honey bee samples and the number of analysed honey bees, this study could be regarded as one of the most dense in terms of *A. mellifera* mtDNA datapoints obtained on average over a geographic area conducted to date. This is in comparison to several other studies carried out in different parts of the world that involved a lower or similar number of honey bees but sampled over larger geographical areas^20–23,25–35,45,59–66^. This high-density map provides a detailed and accurate representation of the distribution of mtDNA haplotypes in the region at a specific time point.

Our data shows that the analysed population includes honey bees carrying mtDNA derived from one of the three main evolutionary lineages, i.e. C, A, M. There is a predominance of 97.6% for the C lineage, within which C1 (the typical mtDNA haplotype of *A. m. ligustica*) was the most frequent mtDNA haplotype (86.6%), followed by C2 (11%). The A and M mtDNA lineages were present among the studied population but with a low cumulative frequency of 2.4%: 1.3% for the A lineage and 1.1% for the M lineage. The population genetic data we gathered from the Emilia-Romagna region offers the opportunity to compare with information from previous studies. However, these other studies^25,48,50^ only partially align with our experimental design in terms of methods utilised, geographic localisation, sample density and time frame of sample collection.

The first distribution map of mtDNA haplotypes based on the same Sanger sequencing approach we utilised was obtained from a study conducted by Franck et al*.*^25^ more than 20 years ago (published in 2000). In their study, Franck et al*.*^25^ analysed honey bees from 18 sampling sites across the Italian Peninsula and the two main Italian islands^25^. However, their study only included one sampling area in the Emilia-Romagna region (Province of Forlì-Cesena), with a total of 38 honey bee samples, resulting in the exclusive presence of the C1 haplotype. This sample may not be representative of the distribution of mtDNA haplotypes in the entire region or even the same province, where we analysed a significantly larger number of honey bees (more than 300 in this province). It is also evident that the population genetic scenarios for *A. mellifera* may have changed over the last two decades, as reported in other European regions^15,34^. Considering the above, it is not surprising that the partial picture of honey bee mtDNA in the Emilia-Romagna region from Franck et al*.*^25^ differs from the results we obtained in the current study.

In two more recent studies that can be used for comparative analyses, the main honey bee mitotypes were identified by using environmental DNA derived from honey, and their distribution was monitored all over Italy, including the Emilia-Romagna region^48,50^. This approach based on the entomological footprint left in the honey can provide a simplified but extensive population genetic analysis, considering that each honey sample may contain the DNA of many different honey bees, derived from different colonies or even different apiaries^48,50^ Therefore, here using the results reported for the Emilia-Romagna region (derived from ~ 100 honey samples produced in all provinces between 2018 and 2020), and combining the findings of these two studies, which initially distinguished only the main mtDNA lineages (A, C and M)^48,50^ and later identified the C1 and C2 haplotypes^48^, the C1 haplotype was confirmed to be the most frequent one. Nearly all honey samples contained the footprint of honey bees carrying the C1 mitotype, which was also the only mitotype identified in 66% of randomly analysed honey samples produced in the Emilia-Romagna region in 2018^48,50^. The proportion of these honey samples that contained only the C2 mitotype was ~ 4% while those with a combination of C1 and C2 mitotypes was ~ 16%^48^. When combined and integrated, these frequencies were similar to the 11% frequency of the C2 haplotype we found in our analysis of individual bees. Other information from the same group of honey samples^48,50^ also revealed the presence of the A and M haplotypes in the Emilia-Romagna region, with ~ 11% and ~ 17% of samples containing mtDNA traces from these lineages, respectively. It is important to note that a single honey sample can contain multiple haplotypes from different colonies, as many bees may have contributed to its production^48,50^. The data from these other studies, which used environmental DNA from honey samples, align more closely with our findings from analysing honey bees directly for mtDNA information than the conclusions drawn by Franck et al*.*^25^. It is interesting to mention that the studies based on honey DNA were conducted around the same time as our study, with honey samples from the Emilia-Romagna region collected in 2018^48,50,51^. Furthermore, the geographic distribution covered by the honey samples^48,50^ is similar to what we obtained in the present study.

We have previously suggested that the unexpected heterogeneity in mtDNA haplotypes found in the Italian honey bee population can be readily explained by beekeepers using hybrid queens, specifically Buckfast hybrids. These hybrids may carry mtDNA haplotypes other than C1 haplotype^66^. Another explanation is the use of non-native *A. mellifera* subspecies^48,50^. These explanations also apply to the specific scenario observed in the Emilia-Romagna region. These hypotheses can also account for similar findings in the honey bee populations in various European countries and areas where the introgression of non-native subspecies or hybrids is compromising the genetic integrity of local ecotypes and subspecies^20,26–28,30,63,67,68^.

In particular, the frequency of foreign A mitotypes that we reported for the Emilia-Romagna region is similar to what was reported in other regions of Europe. For example, Oleska et al*.*^15^ investigated ~ 400 honey bees from colonies sampled in East-Central Europe (including Poland, Hungary and Romania), reporting a frequency of 1.64% for the A mtDNA lineage (including A1e, A4 and A4s haplotypes). In another study, that included ~ 600 honey bees collected in Lithuania, 0.6% had the A4b haplotype^66^. Six A haplotypes were identified in the region of Ile-de-France, around a conservation area for *A. m. mellifera*^68^. In common with these three studies, we identified two A haplotypes (A1e and A4). Three additional A haplotypes (A1a, A26 and A65), which have already been described in other studies^20,21,57^, along with two novel A haplotypes (A2w and A6a), complete the heterogeneity for this lineage in the Emilia-Romagna region, as derived from our study. This study has identified the largest number of different A mitotypes found to date in any European regions outside of the naturally introgressed areas (such as the Iberian Peninsula and Mediterranean Islands, where natural gene flow from nearby African populations has occurred)^20,25,50,69–73^. It is worth noting the geographically and temporally widespread presence of A mitotypes identified in the Emilia-Romagna region. This further supports their human derived origin, likely due to the sporadic utilisation of queens carrying these foreign haplotypes. The identification of rare and peculiar A mitotypes can be viewed from two perspectives. The first perspective is a matter of concern based on the available information. Most of these haplotypes (A1a, A1e, A4, A26 and A65) have been originally detected in Africa and in Africanized bees from South, Central and North America^57,74–80^, raising worries about the introgression of African genes and associated behavioural traits into other populations^15,81^, such as those in the Emilia-Romagna region. The second perspective, closely linked to the first, involves the potential of these rare A-mitotypes (including the two novel A-mitotypes) to trace back their origins. This can be achieved through the implementation of precise monitoring plans for imported or hybrid queens to prevent the spread of non-native genetic pools in the autochthonous *A. m. ligustica* subspecies. Importation from South America, particularly Argentina, where Africanized bees are highly represented^64,74,76^, could be one of the most likely ways for them to be introduced into the Italian population, together with the use of Buckfast queens likely carrying A mitotypes, as previously suggested by others^15,28^. Haplotype A26, which was identified in the Piacenza province, belongs to the A lineage but has unique characteristics. It lacks the complete Q element, only carrying the P sequence, as noted by Chavez-Galarza et al*.*^21^. The complete absence of the Q element in a mitotype from the A lineage is a very rare occurrence, as this architecture is typically seen in *Apis cerana* species, which is the phylogenetically closest species to *A. mellifera*^17,21^.

The frequency obtained for the M haplotypes (1.1%) was lower than that for the A haplotypes. On one hand, this result was somewhat aligned with the findings of Franck et al*.*^25^, who did not report any M haplotypes in their Forlì-Cesena sampling site (the only site in the Emilia-Romagna region they investigated). On the other hand, the result for the M mitotypes was not completely consistent with the possible explanation of the presence of M haplotypes as a consequence of the evolutionary event that considered the Italian Peninsula as a refuge for the M branch during the Quaternary ice period^25^. This is because the M haplotypes we identified were not all from the M7 group (indicated to be ancestrally present in the Italian populations)^25^. Only M3 and M3a, originally reported in *A. m. iberiensis* and included in the M7 group^25^, could potentially be of autochthonous origin in the *A. m. ligustica* subspecies. Haplotype M4, one of the most frequent mitotypes in *A. m. mellifera*^4,19^, could have arrived in the honey bee population of the region in the same way as suggested for the A haplotypes. The origin of another M-haplotype in the analysed honey bee population of the region, namely haplotype M79, is uncertain. This haplotype, not identified by Franck et al*.*^25^ in the Italian population and first reported in *A. m. iberiensis*^20^, contains the P0 element typical of the A lineage but the same restriction sites as the M haplotypes. We believe that M79 has not arrived in the Italian honey bee population in the same way as the haplotype of the M7 group, but rather that it was introgressed very recently, similar to what is suggested for the M4 haplotype. This is supported by its very low frequency (only one sequence was reported) and its identification in the Piacenza Province where several other unique and low frequency haplotypes were detected. Further studies are needed to support this hypothesis.

The greatest concern stems from the relatively high frequency of the C2 haplotype (11%), which is typical of *A. m. carnica*, suggesting that this subspecies has been extensively introduced in the honey bee population of the Emilia-Romagna region. This relatively high frequency may be attributed to the strong adaptation potential of *A. m. carnica*, especially in the Apennine mountains of the region, and the increasing prevalence of the C2 mitotype in hybrid queens of Buckfast origin. It is important to pay closer attention to the C2 mitotype, as it has not been previously documented in the Italian Peninsula except in the contact region of the North-East sector of Italy, near the border with Austria and Slovenia^25^, with a decreasing gradient of this mitotype from North to South^48^.

Finally, it must be considered that the results presented in this study pertain to the mtDNA level and do not offer any direct insights into the extent of introgression at the nuclear genome level nor do they provide any morphometric information. Further studies are required to gather these types of information in regions where legislation regarding the conservation of *Apis mellifera ligustica* is enforced. Additional investigations using whole-genome sequencing data or genotyping with a large number of nuclear DNA markers are necessary to assess the degree of genetic introgression among various honey bee populations^81^. It is also important to monitor the level of adaptation to continental environments and beekeeping systems, as well as evaluate the productive and behavioural characteristics of colonies carrying foreign mitotypes, particularly those of the A lineage, to prevent any inadvertent introduction of Africanized honey bees through trade with South America.

This study offers a thorough and unprecedented analysis of the honey bee population genetics in the Emilia-Romagna region, focusing on mtDNA information. The results are particularly important as this region currently has legislation supporting the breeding and management of *A. m. ligustica* subspecies, which is particularly relevant for the high density of *A. m. ligustica* queen breeders established in the Emilia-Romagna region. Despite analysing a large number of samples, the haplotype diversity was found to be low. This is mainly due to the prevalence of the C1 haplotype, which is characteristic of the *A. m. ligustica* subspecies. This is generally a positive result and should lead to further actions to strengthen the positioning of this subspecies as the prevalent (or unique) genetic pool in the regional territory, as expected from the full application of the regional law. Continuing the genetic monitoring that we started will be important to evaluate the effect of this law at the population genetic level over the coming years. Based on the obtained results, which identified not only *A. m. ligustica* mtDNA haplotypes, we can recommend a more stringent policy to prevent a more extensive diffusion of foreign mitotypes, which might be indicators of erosion of the genetic integrity of the native subspecies of the Emilia-Romagna region, *A. m. ligustica*.

## Supplementary Information

Supplementary Information.

## Data Availability

The sequences of the two identified novel haplotypes are available in EMBL-EBI European Nucleotide Archive (ENA) repository (http://www.ebi.ac.uk/ena) under the project number PRJEB67526, accession numbers OY748522 and OY748523. All data are reported in the text and in the Supplementary material.
