# Supplementary material for: Distribution of honey bee mitochondrial DNA haplotypes in an Italian region where a legislative act is protecting the Apis mellifera ligustica subspecies
Source: Sci Rep. 2024 Sep 4;14:20583. doi: 10.1038/s41598-024-71233-5 (PMC11375103; doi:10.1038/s41598-024-71233-5)
Supplement: Supplementary file 1 — Supplementary Information. [file 41598_2024_71233_MOESM1_ESM.docx]

**Supplementary Material**

**Distribution of honey bee mitochondrial DNA haplotypes in an Italian region where a legislative act is protecting the *Apis mellifera ligustica* subspecies**

Valeria Taurisano, Anisa Ribani, Dalal Sami, Kate Elise Nelson Johnson, Giuseppina Schiavo, Valerio Joe Utzeri, Samuele Bovo, Luca Fontanesi

**Figure S1.** Genetic structure of the tRNALeu-COII region of the 14 mtDNA haplotypes identified in this study, with results of the *in silico* *Dra*I restriction digestion and the position of the variants for the two novel haplotypes (highlighted with an asterisk). Black arrows indicate restriction sites. The *in silico* determined restriction fragment sizes are reported in bp along the P and Q elements for each haplotype.


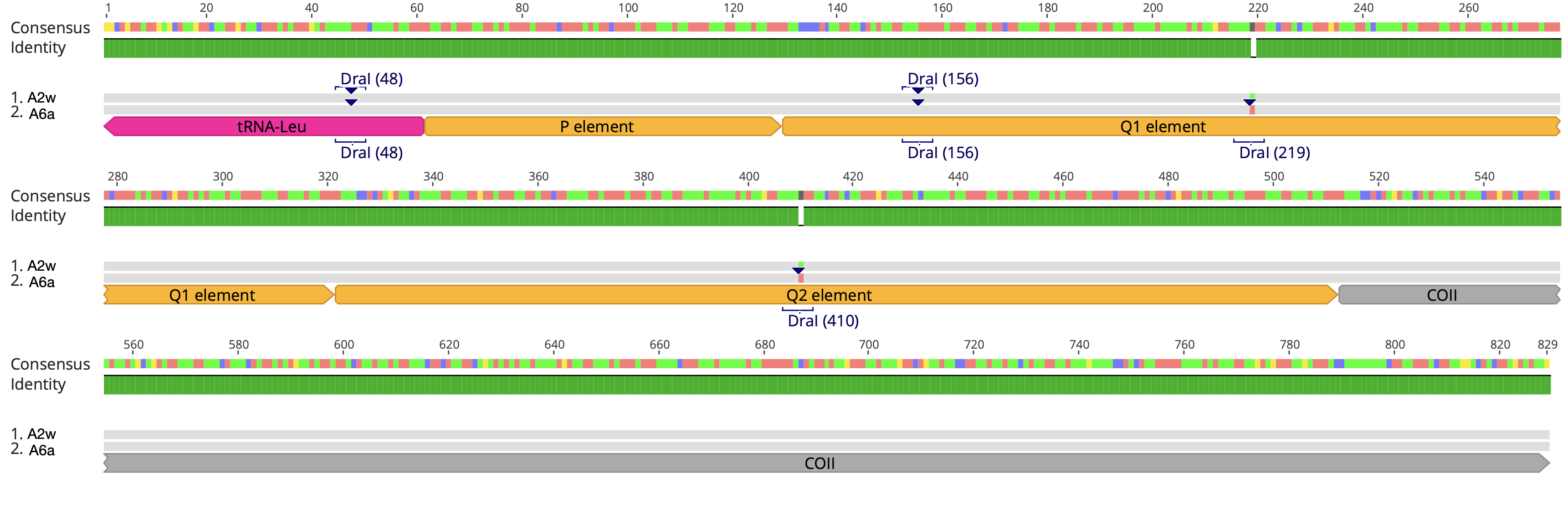


**Figure S2.** Genetic structure of the tRNA-Leu-COII region (with the P0QQ elements highlighted) and in silico *Dra*I restriction digestion results for the two novel mtDNA haplotypes (A2w and A6a).

**Supplementary Table S1.** GenBank/ENA accession numbers and references for *Apis mellifera* mtDNA sequences also identified in this study and for sequences included in the phylogenetic analyses.

| **mtDNA haplotypes** | **Accession numbers^1^** | **References^2^** | **Honey bee subspecies/lines^3^** | **mtDNA haplotypes identified in this study (no. of sequences)** | **mtDNA haplotype used in the phylogenetic analyses^4^** |
| --- | --- | --- | --- | --- | --- |
| A1a | KX463739 | Chávez-Galarza et al. (^20^) | *A. m. intermissa/A. m. iberiensis* | Yes (5) | Yes |
| A1e | MW677198 | Chávez-Galarza et al. (^21^) | N. A | Yes (3) | Yes |
| A2u | KX463769 | Chávez-Galarza et al. (^20^) | *A. m. iberiensis* | No | Yes |
| A4 | EF033650 | Collet et al. (^57^) | Africanized | Yes (2) | Yes |
| A6 | OM994513 | unpublished | *A. m. intermissa* | No | Yes |
| A26 | EF033651 | Collet et al. (^57^) | *Africanized/A. m. adansonii* | Yes (1) | Yes |
| A65 | MW677213 | Chávez-Galarza et al. (^21^) | N. A | Yes (1) | Yes |
| A2w | OY748522 | This study | N. A. | Yes (2) | Yes |
| A6a | OY748523 | This study | N. A. | Yes (1) | Yes |
| M3 | FJ478004 | Franck et al. (^12^) | *A. m. iberiensis* | Yes (1) | Yes |
| M3 | N. A. | Franck et al. (^25^) | *A. m. ligustica* | No | Yes |
| M3a | KX463884 | Chávez-Galarza et al. (^20^) | *A. m. iberiensis* | Yes (8) | Yes |
| M4 | EF033656 | Collet et al. (^57^) | N. A. | Yes (3) | Yes |
| M7 | KX463911 | Chávez-Galarza et al. (^20^) | *A. m. iberiensis* | No | Yes |
| M7a | N. A. | Franck et al. (^25^) | *A. m. iberiensis* | No | Yes |
| M7a | KF274639 | Pinto et al. (^30^) | *A. m. ligustica* | No | Yes |
| M7b | N. A. | Franck et al. (^25^) | *A. m. ligustica* | No | Yes |
| M7b | MW677214 | Chávez-Galarza et al. (^20^) | N. A. | No | Yes |
| M7c | OM107914 | unpublished | N. A. | No | Yes |
| M27 | N. A. | Franck et al. (^25^) | *A. m. ligustica* | No | Yes |
| M79 | KX463882 | Chávez-Galarza et al. (^20^) | *A. m. iberiensis* | Yes (1) | Yes |
| C1 | NC_001566 / MW677216 | Chávez-Galarza et al. (^21^) | *A. m. ligustica* | Yes (989) | Yes |
| C2c | MN_250878.1 / MW677217 | Chávez-Galarza et al. (^21^) | *A. m. carnica* | Yes (2) | Yes |
| C2 | NC_061380.1 / MW677218 | Chávez-Galarza et al. (^21^) | *A. m. carnica* | Yes (124) | Yes |

^1^ Accession number was not available (N. A.) for sequences reported in Franck et al. (^25^). Sequences were retrieved from Figure 2 of that publication.

^2^ References that reported the sequences that were also identified in this study or that were used in the phylogenetic analyses (with the corresponding accession number of source of the sequence information).

^3^ *A. mellifera* subspecies or lines from which the indicated sequences were reported, based on the information available in the corresponding references and accession numbers.

^4^ Some sequences not identified in this study were included in the phylogenetic analyses to provide meaningful information on the phylogenetic position of the mtDNA haplotypes.

**Supplementary Table S2.** Distribution of the analysed honey bees over year of sampling and administrative province, with details on the number of the identified mtDNA haplotypes. The number of the specific haplotypes (indicated within the brackets) is reported for the A and M lineages.

| **Province/Year** | **2020** | | | | | **2021** | | | | | **2022** | | | | |
| --- | --- | --- | --- | --- | --- | --- | --- | --- | --- | --- | --- | --- | --- | --- | --- |
|  | C1 | C2 | M | A | Total | C1 | C2 | M | A | Total | C1 | C2 | M | A | Total |
| Piacenza (PC) | 10 | 2 | 0 | 1 (A6a) | 13 | 32 | 12 | 1 (M3a) | 3 (2 A2w, 1 A26) | 48 | 96 | 17 | 1 (M79) | 1 (A65) | 115 |
| Parma (PR) | 15 | 2 | 1 (M3a) | 1 (A1a) | 19 | 94 | 9 | 2 (2 M3a) | 2 (2 A1e) | 107 | 76 | 8 | 2 (2 M3a) | 1 (A1e) | 87 |
| Reggio Emilia (RE) | 5 | 0 | 0 | 0 | 5 | 33 | 2 | 1 (M4) | 0 | 36 | 23 | 2 | 0 | 0 | 25 |
| Modena (MO) | 3 | 0 | 0 | 0 | 3 | 29 | 1 | 0 | 0 | 30 | 0 | 0 | 0 | 0 | 0 |
| Bologna (BO) | 24 | 3 | 2 (1 M3, 1 M4) | 0 | 29 | 63 | 1 | 1 (M4) | 0 | 65 | 54 | 2 | 0 | 0 | 56 |
| Ferrara (FE) | 11 | 0 | 0 | 0 | 11 | 0 | 0 | 0 | 0 | 0 | 5 | 0 | 0 | 0 | 5 |
| Ravenna (RA) | 4 | 0 | 0 | 0 | 4 | 58 | 8 | 0 | 1 (A1e) | 67 | 4 | 2 | 0 | 0 | 6 |
| Forlì – Cesena (FC) | 3 | 0 | 0 | 0 | 3 | 141 | 24 | 1 (M3a) | 0 | 166 | 121 | 18 | 1 (M3a) | 5 (3 A1a, 2 A4) | 145 |
| Rimini (RN) | 5 | 2 | 0 | 0 | 7 | 61 | 8 | 0 | 0 | 69 | 19 | 3 | 0 | 0 | 22 |
| Total | 80 | 9 | 3 | 2 | 94 | 511 | 65 | 6 | 6 | 588 | 398 | 52 | 4 | 7 | 461 |
